# Epigenetics of Bladder Cancer: Where Biomarkers and Therapeutic Targets Meet

**DOI:** 10.3389/fgene.2019.01125

**Published:** 2019-11-18

**Authors:** Victor G. Martinez, Ester Munera-Maravilla, Alejandra Bernardini, Carolina Rubio, Cristian Suarez-Cabrera, Cristina Segovia, Iris Lodewijk, Marta Dueñas, Mónica Martínez-Fernández, Jesus Maria Paramio

**Affiliations:** ^1^Biomedical Research Institute I + 12, University Hospital 12 de Octubre, Madrid, Spain; ^2^Molecular Oncology Unit, CIEMAT (Centro de Investigaciones Energéticas, Medioambientales y Tecnológicas), Madrid, Spain; ^3^Centro de Investigación Biomédica en Red de Cáncer (CIBERONC), Madrid, Spain; ^4^Genomes & Disease Lab, CiMUS (Center for Research in Molecular Medicine and Chronic Diseases), Universidade de Santiago de Compostela, Santiago de Compostela, Spain

**Keywords:** Epigenetic, chromatin remodelling, bladder cancer, biomarkers, therapeutic target

## Abstract

Bladder cancer (BC) is the most common neoplasia of the urothelial tract. Due to its high incidence, prevalence, recurrence and mortality, it remains an unsolved clinical and social problem. The treatment of BC is challenging and, although immunotherapies have revealed potential benefit in a percentage of patients, it remains mostly an incurable disease at its advanced state. Epigenetic alterations, including aberrant DNA methylation, altered chromatin remodeling and deregulated expression of non-coding RNAs are common events in BC and can be driver events in BC pathogenesis. Accordingly, these epigenetic alterations are now being used as potential biomarkers for these disorders and are being envisioned as potential therapeutic targets for the future management of BC. In this review, we summarize the recent findings in these emerging and exciting new aspects paving the way for future clinical treatment of this disease.

## Introduction

BC is a common urogenital cancer which represents a current clinical and social problem. At diagnosis, two thirds of patients present a non-muscle invasive bladder cancer (NMIBC), a relatively limited aggressive disease confined to the bladder and without signs of invasion of the underlying muscle layer. The remaining patients display muscle-invasive bladder cancer (MIBC) (Knowles and Hurst, 2015). This pathological classification also defines clinical management. NMIBC is treated by transurethral resection, which can be followed by intravesical instillation with Bacillus Calmette–Guérin (BCG) or mitomycin (Babjuk et al., 2017). However, a large proportion (60–75%) of NMIBC patients relapse and, in some cases (15–25%), the recurrent tumor shows signs of MIBC indicating tumor progression (van Rhijn et al., 2009). The current therapeutic options for MIBC include radical cystectomy and platin-based chemotherapy in adjuvant or neoadjuvant settings (Stenzl et al., 2011). However, in a high proportion of cases, the disease progresses showing metastatic spread, which is associated with extremely low survival rates (Stenzl et al., 2011; Pal et al., 2013; Witjes et al., 2014a). No major improvement in MIBC management occurred during the last decades, until recent years, in which immunotherapy has been shown to increase survival with responses in 20–30% of the patients presenting advanced and metastatic BC (Powles et al., 2014; Rosenberg et al., 2016; Balar et al., 2017; Bellmunt et al., 2017; Plimack et al., 2017). As in other cancers, immunotherapy in BC is mainly based on the use of antibodies that prevent PD-1/PD-L1 interaction, the so called immune checkpoint, leading to immune killing of tumor cells (Pardoll, 2012). The limited activity of immune checkpoint inhibitors in the clinics has led to the consideration of possible combinations of different immune and non-immune therapies (Gotwals et al., 2017). Moreover, in the case of BC patients, it is unclear which patients are more likely to benefit from this treatment (Powles et al., 2014; Rosenberg et al., 2016; Balar et al., 2017; Bellmunt et al., 2017; Plimack et al., 2017). Thus, there is a need not only for more effective therapies in patients with advanced BC, but also for new biomarkers that will help to define which patients may benefit from immunotherapy (Havel et al., 2019).

Epigenetics are heritable but reversible modifications that alter gene expression without changing primary DNA sequences. Epigenome functions are fundamental for the normal status of gene expression and their alterations affect basic cellular processes such as proliferation, differentiation and apoptosis, which may lead to important diseases including cancer (Liep et al., 2012; Baylin and Jones, 2016). Therefore, epigenetic-based cancer biomarkers are promising tools for detection, diagnosis, assessment of prognosis, and prediction of response to therapy (Esteller, 2008; Jerónimo and Henrique, 2014). An extraordinary number of alterations in epigenetic machinery have been observed in BC, affecting DNA methylation (Marques-Magalhaes et al., 2018), chromatin organization, histone modifications (Weinstein et al., 2014; Robertson et al., 2018) and non-coding RNAs expression (Pop-Bica et al., 2017; Taheri et al., 2018). This has produced a large body of evidence indicating that epigenetic machinery could represent a putative target for BC management, a source of valuable biomarkers for diagnostic, prognostic and response prediction, and also a novel research field with amazing new insights into the molecular mechanisms of cancer biology governing cell autonomous cancer processes as well as the intricate cross talk between cancer cells and their niche.

## Chromatin Remodelers in Bc

The epigenome is defined by changes that do not involve alterations in the DNA nucleotide sequence. These changes are broadly divided into DNA methylation and modifications of the histone tails that allow the opening or closing of the chromatin. The functions of the epigenome are fundamental for normal gene expression, and its alterations affect basic cellular processes (Tsai and Baylin, 2011; Liep et al., 2012). The aberrant epigenetic landscape is a hallmark of human cancer (Han et al., 2012; Mio et al., 2019; Zhao et al., 2019) and, in particular, characterizes BC as an epigenome disease, as studies of complete exome sequencing have shown that it presents frequent alterations in the genes that govern the organization of chromatin and histone modifications, either by mutation or by its expression/altered function (Gui et al., 2011; Weinstein et al., 2014).

**Figure 1:**
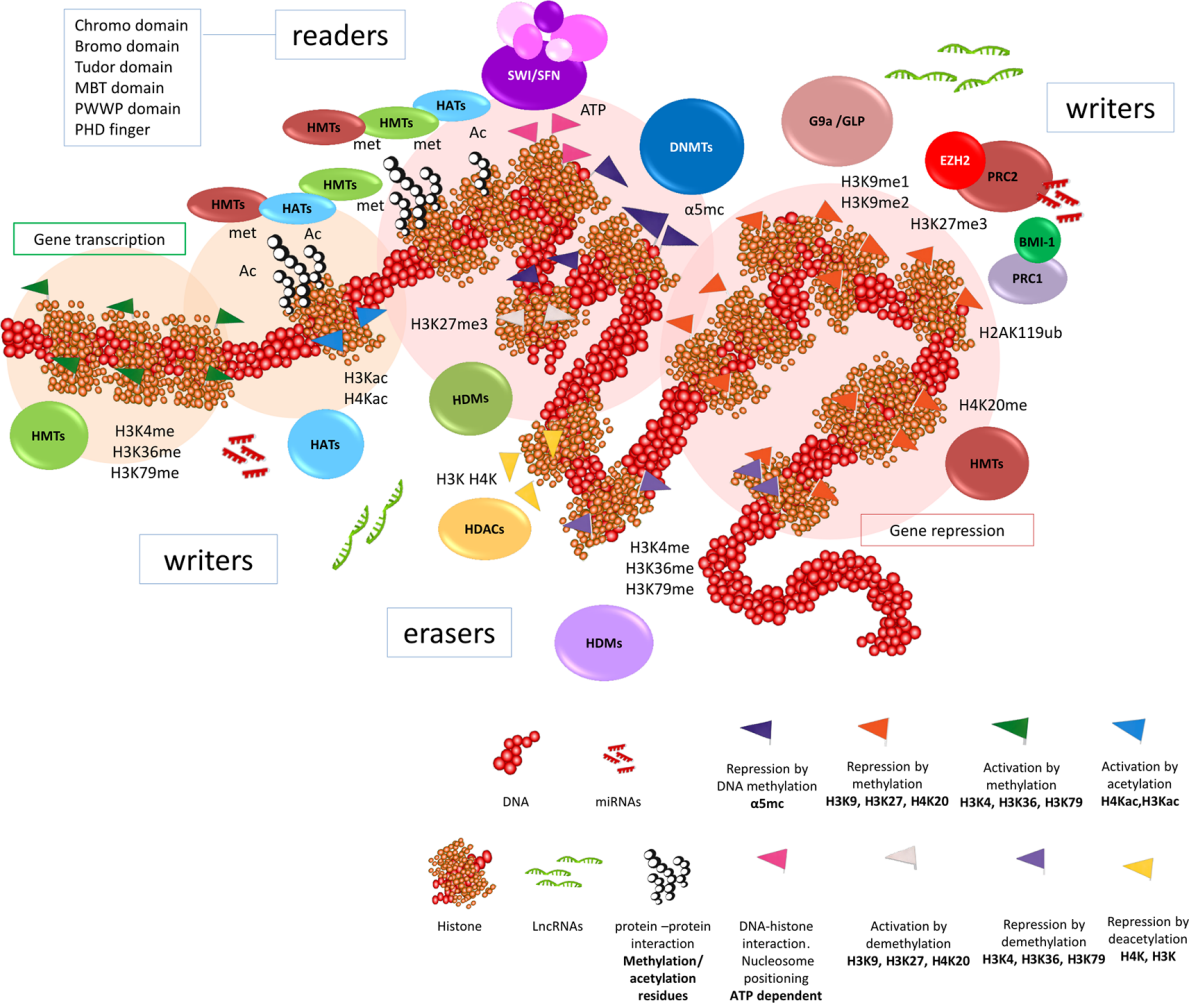
Epigenetic regulation in cancer cells. General scheme of the dynamic interaction of DNA methylation, histone modifications, positioning of nucleosomes, among other factors, that participate in the mechanisms of the epigenome to regulate gene expression. Thus, the tumor cell acquires a particular identity. DNA methylation is present throughout the genome, however we can find aberrant DNA methylations or alterations in the DNMTs enzymes (methyltranferases of DNA nucleotides) in the tumor. The methylation mark H3K27 is the main brand that controls the gene repression in euchromatin. The remodeling enzymes called *writers* (HMT, histone methyltransferase, HAT, histone acetyltransferase), *erasers* (HDM, histone demethylase; HDAC, histone deacetylase) and *readers* (specialized interaction motif containing proteins that recognize post-translational modifications, mostly acetylation and methylation) of the main histone modifications work in a coordinated manner for the regulation of gene transcription. Depending on the genes they regulate, they are recruited to the same place to function together. Therefore, all these molecules are subject of study as possible therapeutic targets.

Nevertheless, the mechanisms for epigenetic regulation of gene expression are not limited to chromatin modifiers or DNA methylation changes, as non-coding RNAs are also involved (Fabbri and Calin, 2010; Gupta et al., 2010; Kogo et al., 2011) (**Figure 1**).

### DNA Methylation in BC

Methylation of DNA is the process by which a methyl group is added by a covalent bound to the 5’ position of a cytosine ring of the DNA molecule. The methylation event is a frequent epigenetic episode and usually occurs on a cytosine followed by a guanine (CpG dinucleotide). There are regions of the genome, termed CpG islands, which contain a higher density of the CpG dinucleotide than the rest of the genome (Li et al., 2016a). These CpG islands are located in sites that normally overlap with gene regulatory regions (Baylln et al., 1997). Thereupon, there are CpG islands at promoter/5’ regions of 50% of all known genes and they are normally unmethylated (Reinert, 2012) which is associated with (potentially) active transcription (Jones and Liang, 2009). CpG islands are also found in gene bodies and their methylation status positively correlates with gene expression (Yang et al., 2014). DNA methylation is a key process in mammalian development, and its alterations are hallmarks of diseases, including cancer. Changes in normal DNA methylation status exist in approximately 50–90% of BCs, including DNA hypermethylation of promoter sites of *A3BP1*, *NPTX2*, *ZIC4*, *PAX5A*, *MGMT*, *IGSF4*, *GDF15*, *SOX11*, *HOXA9*, *MEIS1*, *VIM*, *STK11*, *MSH6*, *BRCA1*, *TBX2*, *TBX3*, *TERT*, *GATA2*, *DAPK1*, *CDH4*, *CCND2*, *GSTP1*, *CDKN2A*, *CDKN2B*, *WIF1*, *RASSF1A*, among others (Porten, 2018). These genes are mainly tumor suppressors that belong to biological pathways such as DNA repair, cell cycle control, cell invasion and apoptosis (Reinert et al., 2011; Sánchez-Carbayo, 2012). DNA methylation of promoter regions typically negatively affects gene expression, which can promote the development (Costa et al., 2010; Chung et al., 2011) and progression of BC (Yates et al., 2007; Kandimalla et al., 2012; Casadevall et al., 2017), and can predict therapy outcomes (Agundez et al., 2011; Xylinas et al., 2016).

First studies of DNA methylation in BC focused on potential genes which methylated status might correlate with stage, grade and recurrence. More recently, the development of modern whole-genome DNA methylation assays has allowed to analyze in depth the BC methylome. Wolff et al. stablished that most DNA methylation changes happen in early BC and are conserved in carcinoma *in situ*, non-invasive as well as invasive tumors, and are located in CpG islands (Wolff et al., 2010). Furthermore, the degree and extent of hypermethylation correlates with grade and stage since low-grade tumors have less altered methylation loci compared to high-grade and invasive tumors (Catto et al., 2005; Yates et al., 2007; Wolff et al., 2010). DNA methylation also separates mutation status of *FGFR3*. NMIBC *FGFR3* wild-type tumors, which have a poorer prognosis compared to *FGFR3* mutant NMIBC (Van Rhijn et al., 2012), were more methylated than *FGFR3*-mutant tumors (Serizawa et al., 2011; Kandimalla et al., 2012). Besides, in low-grade non-invasive tumors, DNA hypomethylation was more frequent than in invasive tumors (Wolff et al., 2010). Hypermethylation of *ZO2*, *MYOD* and *CDH13* was also detected in normal-appearing urothelium from bladder with cancer compared to urothelium from healthy bladder, indicating an epigenetic ‘field defect’ and a possible contribution to a loss of epithelial integrity, likely generating a permissive environment for tumor recurrences (Wolff et al., 2010; Majewski et al., 2019).

Since several genes were identified as frequently hypermethylated in primary BC, diagnosis could be performed based on the methylated status of a gene set. For instance, methylation of *IPF1*, *GALR1*, *TAL1*, *PENK* and *TJP2* was found to be higher in MIBC tumors than in NMIBC (Wolff et al., 2010). Sacristan et al. indicated that methylation of *RARB*, *CD44*, *GSTP1*, *IGSF4*, *CHFR*, *PYCARD*, *TP53*, *STK11* and *GATA5* distinguished low-grade versus high-grade tumors, whereas Olkhov-Mitsel et al. stablished that the inclusion of *GP5* and *ZSCAN12* in a methylation panel could feasibly distinguish high-grade and low-grade BC (Olkhov-Mitsel et al., 2017). Unluckily, the overlap between genes found in different studies is limited.

Since 20% of BC patients recur, finding epigenetic markers of progression would be useful to predict recurrence. A wide study reviewed 87 articles reporting the association of epigenetic markers with prognostic outcomes (Casadevall et al., 2017). However, the prognostic influence of epigenetic alterations in BC remains unclear. *CACNA1G* (García-Baquero et al., 2014) and *TBX3* (Kandimalla et al., 2012) were associated with progression and *SFRP5* correlated with recurrence (García-Baquero et al., 2014). *CDNK2A* is methylated in 64% of BCs, however, inconsistent results were found in prognosis (Casadevall et al., 2017). Based on TCGA data, methylation and expression levels of *SOWAHC* were found to be correlated with prognosis (Yang et al., 2019). *HOX* genes appear hypermethylated in almost all aggressive tumors (Reinert et al., 2011; Kandimalla et al., 2012), and *HOXA9* promoter methylation correlated with higher recurrence, progression, and death by cancer in NMIBC and MIBC (Kitchen et al., 2015) and was associated with cisplatin resistance in BC cell lines (Xylinas et al., 2016). High-risk NMIBC manifest higher rates of progression to invasive tumors than low- and intermediate-risk bladder tumors, which in many cases do not recur or progress. Recently, some investigations proposed multiple CpG sites differentially methylated between high-risk recurrence/progression tumors and less aggressive low-risk no-recurrence tumors (Kitchen et al., 2018; Peng et al., 2018).

A three-gene methylation panel which differentiates between patients with metastatic and free of cancer lymph nodes might also be predictive of metastasis development, and enable the selection of patients that would benefit from lymph node resection and neoadjuvant chemotherapy (Stubendorff et al., 2019). In patients undergoing BCG treatment, methylation status of *MSH6* and *THBS1* may help to distinguish responders to therapy, and methylation of *GATA5* associated with survival (Agundez et al., 2011), allowing the possible identification of patients requiring a more aggressive therapy. After chemotherapeutic treatment, the *MDR1* gene was found to be overexpressed in BC compared with untreated tumors, and in tumors from patients that eventually recurred. This overexpression correlated negatively with methylation of CpG sites in the promoter region (Tada et al., 2000). An interesting study tested gene methylation in second recurrences in bladder of primary upper-tract urothelial carcinomas, and stablished that the methylation rate in certain genes tend to increase with the number of recurrences, which may be a predictive factor for recurrences after surgery (Guan et al., 2018). Nevertheless, the existence of inconsistent results and lack of validation studies hampers at present relevance of these findings (Casadevall et al., 2017; Porten, 2018).

Less data is reported about hypomethylation status in BC. In 1983, a pioneer study reported that hypomethylation could distinguish genes of cancer cells compared with their normal counterparts (Feinberg and Vogelstein, 1983). In normal cells, certain CpG rich satellite repeats are strongly methylated, such as LINE-1 (Schulz, 2006). Interestingly, these regions are strongly hypomethylated in all types of BC (Kreimer et al., 2013) and could translate in genomic instability (Wolff et al., 2010). Besides, as a tissue-fingerprint, the hypomethylation pattern of LINE-1 seems to be specific for each tumor type and tissue (Sharma et al., 2019). Furthermore, a different type of study analyzed global methylation in DNA from blood cells and found that leukocyte DNA hypomethylation is a risk factor for BC (Moore et al., 2008).

DNA methylation is catalyzed by three DNA methyltransferases (DNMT): DNMT1, DNMT3A and DNMT3B. DNMT1 is the keeper of the regular methylation status of the genome after cell replication (Goll and Bestor, 2005), whereas DNMT3a and DNMT3b are *de novo* methyltransferases (Okano et al., 1999). Mutations in chromatin regulatory genes are present in around 76% of BC (Robertson et al., 2018) and are more frequently found in BC than in any other solid tumor (Weinstein et al., 2014). However, the alterations regarding DNMTs in BC are mainly found to be an increase in their expression (Li et al., 2016a). Several genes that are methylated in BC are repressed by polycomb complexes (Wolff et al., 2010; Kandimalla et al., 2012). These complexes composed of EZH2 recruit DNMTs required for DNA methylation (Viré et al., 2006), which suggests an upstream regulation of methylation in BC.

### Chromatin Remodeling and Histone Modification in BC

Mutations in chromatin remodeling genes are very frequent in BC (Robertson et al., 2018), affecting 89% of histone remodelers and 64% of nucleosome positioning genes in MIBC (Weinstein et al., 2014; Robertson et al., 2018). The post-translational modifications of histones, such as acetylation, methylation, phosphorylation or ubiquination in specific residues of lysines, arginines and serines (Allis et al., 2007; Rothbart and Strahl, 2014), modulate the dynamic and reversible changes in chromatin structural changes. This “histone code” can be written, erased and read by different molecules modulating transcription (Gillette and Hill, 2015). Therefore, chromatin remodelers can be classified as writers (methyltransferases (HMTs) or acetylases (HATs)), erasers (demethylases (HDMs) and deacetylases (HDACs)) and readers, which are further divided in proteins or effector complexes that interact with specific domains (Gillette and Hill, 2015; Hyun et al., 2017), and nucleosome remodeling multiprotein complexes that are able to alter DNA-histone contacts. The main marks of gene transcription are acetylation of histone 3 and histone 4 (H3Kac, H4Kac) and methylation of histone 3 on lysine 4, 36 and 79 (H3K4me, H3K36me, H3K79me), while methylation of histone 3 on lysine 9 and 27 and histone 4 on lysine 20 (H3K9me, H3K27me, H4K20me) represent important marks for gene repression (Bernstein et al., 2007) (**Figure 1**).

#### Writers

Histone methyltransferase EZH2 catalyzes H3K27me2 and H3K27me3 marks to regulate the repression of gene expression (Deb et al., 2014), and compacts chromatin with other molecules like BMI-1 (Cao et al., 2005) (**Figure 1**). Its involvement in tumor development and progression is a common characteristic of several human tumors, including BC (Yamaguchi and Hung, 2014). It has been demonstrated that the existence of the oncogenic axis Rb-E2F-EZH2 predicts recurrence and progression in NMIBC (Santos et al., 2014) and promotes global changes in gene expression, including the aberrant expression of lncRNAs such as *HOTAIR* (Martínez-Fernández et al., 2015b), and the silencing of several microRNAs, such as mir-200 family (Martínez-Fernández et al., 2015a). Several studies have shown that EZH2 also interacts with other modifiers such as DNMTs, HDAC or G9a, that could explain some oncological properties of EZH2. The importance of these non-canonical functions of EZH2 in BC is still not well understood, although it could favor intratumoral heterogeneity (Gupta et al., 2011).

Histone methyltransferase G9a (EHMT2) is considered an oncogenic epigenetic factor (Lee et al., 2015), which can be involved in urothelial tumors (Shankar et al., 2013; Cho et al., 2015). This enzyme binds GLP (EHMT1) and catalyzes H3K9me2 leading to gene silencing through physical interaction with cofactors (Bian et al., 2015; Maier et al., 2015; Simon et al., 2015; Hu et al., 2018) and/or non-coding RNAs (Nagano et al., 2008). Additionally, G9a may interact with EZH2 allowing the silencing of specific loci in a cooperative way (Mozzetta et al., 2014; Mozzetta et al., 2015) becoming a possible target for advanced metastatic BC (Segovia et al., 2019).

Methyltransferase KMT2D (MLL2), which catalyzes H3K4me1 and H3K4me2 (Lee et al., 2013), displays the highest mutation rate among all HMTs in BC (Weinstein et al., 2014) in close association with tumor development, recurrence (Wu et al., 2016a) and resistance to therapy (Lu et al., 2017). *KMT2C* (MLL3) is also commonly mutated in high grade NMIBC (Weinstein et al., 2014; Hurst et al., 2017) and in luminal papillary and basal squamous MIBC subtypes (Robertson et al., 2018), and its silencing affects DNA damage response genes (Rampias et al., 2019). Additionally, somatic mutations of 13 HMT genes, including *NSD1* and *NSD3,* are present in a high proportion of BC tumors (Ding et al., 2019). Moreover, the genes encoding acetyltransferases EP300 and CREBBP are among the genes most frequently inactivated by mutation in human BC (Gui et al., 2011; Duex et al., 2018b).

#### Erasers

The gene encoding histone demethylase KDM6A (UTX), located on the X chromosome, is one of the genes most frequently mutated in BC (Gui et al., 2011; Nickerson et al., 2014). This demethylase can specifically erase the marks written by EZH2 (Agger et al., 2007; Lee et al., 2007). Mutations in *KDM6A* are more common in NIMBC and in women (Hurst et al., 2017), and tend to be mutually exclusive with *MLL2* alterations (Kim et al., 2015a) suggesting a predominant silenced chromatin during bladder carcinogenesis (Casadevall et al., 2017). In some cases, it has been associated with *RB1* mutation in high grade urothelial tumors (Balbás-Martínez et al., 2013; Ross et al., 2014).

Acetylation of lysine residues in histone tails results in a more open state of the chromatin (Roger et al., 2011) and histone acetylation levels decrease during progression towards MIBC (Ellinger et al., 2016). Furthermore, the deregulated expression of various HDACs, like HDAC1, 2, 3 and 6, has been described in urothelial tumors in close association with malignancy (Chen et al., 2011; Li et al., 2016b; Niegisch et al., 2013; Poyet et al., 2014; Lee and Song, 2017).

#### Readers

The effects of epigenetic marks are mediated through effector complexes which “read” marks and facilitate the DNA–histone and protein–protein interactions. This provides recruitment platforms for other epigenetic regulators to specific DNA loci (Dawson and Kouzarides, 2012) (**Figure 1**). The methylation and acetylation writers usually have reader domains (predominantly bromodomain (BRD) and plant homeodomain (PHD) finger) that allow recognition of the histone methylation/acetylation status (Dawson and Kouzarides, 2012; Biswas and Rao, 2018).

Methyl CpG sites are recognized by proteins that contain conserved binding domains such as methyl CpG binding domain (MBD), SRA domain and zinc finger (ZnF). These proteins work together with other factors to alter the transcriptional status of DNA (Biswas and Rao, 2018). The histone methylated residues are recognized by conserved binding domains such as PHD finger, Tudor domain, PWWP (Pro-Trp-Trp-Pro) domain, chromodomain, malignant brain tumor domain (MBT), ankyrin repeats (present in G9a and GLP1), ZnFs and WD40 domain, among others. Furthermore, BRDs, double PHD finger and Yeats domains bind specifically to acetylated residues of histones (Dhalluin et al., 1999; Fischle, 2003; Kouzarides, 2007; Taverna et al., 2007; Dawson and Kouzarides, 2012; Biswas and Rao, 2018). BRDs are present in the acetylation writers CBP and p300 along with several protein interaction motifs, both closely related proteins have been deeply investigated since they are able to acetylate the four histones (Dawson and Kouzarides, 2012). Additionally, BRDs of chromatin remodeling enzymes BRM (*SMARCA2*) and BRG1 (*SMARCA4*) recognize multiple acetylation sites at H3 and H4. In BC, the BRD4 histone acetylation reader is overexpressed and can upregulate C-MYC, which controls the expression of cell cycle progression genes, enhancing the recruitment of this factor to the EZH2 promoter and subsequently upregulating EZH2 expression, which has a significant relevance on tumor growth (Wu et al., 2016b). Consequently, EZH2 promotes growth of BC by chromatin modification (Wu et al., 2016b), especially in tumors with loss of *KDM6A* (Ler et al., 2017). Some susceptibilities to EZH2 inhibitors have been found in relation to mutations in components of SWI/SNF complexes such as *ARID1B* (12%), *SMARCA4* (15%) *SMARCA2* (16%) (Helming et al., 2014; Bitler et al., 2015; Kim et al., 2015b). This is relevant in the context of BC, since components of the SWI/SNF complexes are also frequently altered in BC patients (Knowles and Hurst, 2015; Robertson et al., 2018). Other remodelers such as the SWI/SNF nucleosomal complex component, *ARID1A*, often show inactivating mutations or deep eliminations in both MIBC (Weinstein et al., 2014; Robertson et al., 2018) and NIMBC (Hurst et al., 2017).

An additional complexity of chromatin remodeling lies in the fact that many chromatin regulators have more than one type of reader domain, and their binding to chromatin can be further influenced by histone modifications (Ruthenburg et al., 2007). The understanding of the dynamic plasticity of DNA and histone modifications will allow us to open new venues to the management and treatment of BC.

### Non-Coding RNAs in BC Etiology and Progression

Non-coding RNAs (ncRNA) represent an important role in the epigenetic changes leading to BC development and progression. Additional to transfer RNA and ribosomal RNA molecules, which represent the most abundant ncRNAs (3–10 × 10^7^ and 3–10 × 10^6^ molecules per cell, respectively), several ncRNA classes can be distinguished, including long non-coding RNA (lncRNA), transcribed ultraconserved region (T-UCR), circular RNA (circRNA), small interfering RNA (siRNA), Y RNA (Y RNA), micro-RNA (miRNA; miR), piwi-interacting RNA (piRNA), small nucleolar RNAs and small nuclear ribonucleic acid (Palazzo and Lee, 2015; Anastasiadou et al., 2017; Gulìa et al., 2017)

NcRNA molecules are specific RNAs which are not translated into proteins, and represent essential regulatory roles in practically every aspect of cellular function. They have been suggested to exert an essential function in the maintenance of genomic stability, mainly through adjusting DNA expression and complex formation with other ncRNA molecules as well as proteins. Consequently, the description of ncRNA function in isolation is very complicated. Several ncRNAs (like miRNAs) are able to target the messenger RNAs (mRNAs) of multiple other genes, whereas the mRNA of one gene can also be targeted by numerous miRNAs. Furthermore, miRNAs can interact with other ncRNA molecules, like lncRNAs and circRNAs, in order to control their stability, while lncRNAs and circRNAs are able to regulate the abundance of miRNAs. Besides, ncRNAs can interact with individual proteins and protein complexes which might facilitate specific protein targeting or the assembly of protein complexes by providing a scaffold (Anastasiadou et al., 2017; Gulìa et al., 2017).

LncRNAs and miRNAs represent the two main classes of ncRNA involved in BC epigenetic etiology as well as progression, and will be discussed in detail below. Additionally, some other ncRNA molecules associated to this pathology will be briefly described.

#### Long Non-Coding RNAs

LncRNAs consist of more than 200 nucleotides, and are involved in several essential biochemical processes (Wang and Chang, 2011). Clark et al. examined about 7,200 lncRNA molecules and described a wide variation in stability, ranging from half-lives of less than 30 min for unstable molecules to half-lives of more than 48 h for extremely stable lncRNAs, with a median lncRNA half-life of 3.5 h (Clark et al., 2012). Besides, these lncRNA molecules have been found to be significantly less abundant than, for example, total mRNA (3–50 × 10^3^ versus 3–10 × 10^5^ molecules per cell, respectively) (Palazzo and Lee, 2015). Many lncRNAs were found to be differentially expressed in a wide range of tumor tissues compared to corresponding healthy control tissues, suggesting an important role in carcinogenesis (Martens-Uzunova et al., 2014; Bhan et al., 2017). In BC, deregulation of lncRNAs has been found to contribute to carcinogenesis in several ways including sustained proliferative signaling and induction of invasion as well as metastasis (Bhan et al., 2017).

LncRNA expression in BC has been extensively reviewed (Gulìa et al., 2017; Taheri et al., 2018). Based on their expression patterns and functions in BC tissue compared to healthy control tissue, lncRNA molecules can be classified in two groups, either showing increased (oncogenic lncRNAs) or decreased (tumor suppressor lncRNAs) expression in tumor tissue. For example, oncogenic lncRNA-*UCA1* has been reported to induce epithelial-mesenchymal transition (EMT) and promote BC cell migration and invasion through the miR-145–ZEB1/2–FSCN1 pathway, as well as by targeting miR-582-5p or modulation of the miR-143/HMGBG1 signaling pathway (Xue et al., 2016; Luo et al., 2017; Wu et al., 2019a). Overexpression of *UCA1* has been associated with high risk of poor outcome in BC. Accordingly, the use of *UCA1* as potential biomarker is subject of ongoing research (Wang et al., 2006; Cui et al., 2017). LncRNA-*H19* has been found to be abundantly expressed in BC leading to increased miR-675 expression, thus inhibiting TP53 activation (Ariel et al., 2000; Liu et al., 2016). LncRNA-*H19* has further been described to promote metastasis and EMT through E-cadherin inhibition as well as by targeting miR-29b-3p (Lv et al., 2017; Zhu et al., 2018). Other well-described oncogenic lncRNAs involved in BC include *MALAT1*, *HOTAIR*, *TUG1*, *ANRIL* and *PVT1*, whereas well-known lncRNAs-*MEG3* and *GAS5* represent tumor suppressor lncRNA molecules (Sun et al., 2015; Gulìa et al., 2017; Guo et al., 2018; Liu et al., 2017b; Xie et al., 2017a; Yang et al., 2017; Yu et al., 2019; Jiao et al., 2018; Liu et al., 2018a; Wang et al., 2018b; Huang et al., 2019a; Tian et al., 2019). Even though many other oncogenic and tumor suppressor lncRNAs have recently been identified in BC, they need further investigation to validate their relevance in this disease. Additionally, the use of specific lncRNA as biomarkers or therapeutic targets is subject of ongoing research and will be further discussed below.

#### Micro-RNAs

As abovementioned, lncRNAs extensively interact with miRNA in the regulation of oncogenic pathways. MiRNAs consist of 21–24 nucleotides and play important roles in the regulation of gene expression (Sohel, 2016). Mature miRNAs have shown high stability reflecting half-lives of approximately 8 hours in the cell, which is reflected in a relatively high abundance of miRNA molecules (1–3 × 10^5^ molecules per cell) (Palazzo and Lee, 2015).

Aberrantly expressed miRNAs have been found in BC tissues causing an altered expression of target genes, resulting in BC development and progression (Zhu et al., 2011). As for lncRNAs, miRNA expression in BC has been extensively reviewed (Enokida et al., 2016; Gulìa et al., 2017). The aberrant expression of several miRNAs has been found to alter two main genetic pathways predisposing to BC. Some miRNAs target the FGFR3 pathway (including miR-99a, miR-100, miR-101, and miR-145), while other miRNA molecules modify the TP53 pathway (such as miR-21 and miR-373) (Homami and Ghazi, 2016). Like lncRNAs, miRNA molecules can be divided in oncogenic miRNAs or tumor suppressor miRNAs. For example, the decreased expression of miR-34a in BC has an anti-metastatic function through the CD44/EMT signaling pathway (Yu et al., 2014) and through targeting NOTCH1 and HNF4G also negatively modulates BC cell proliferation and invasion (Zhang et al., 2012; Sun et al., 2015). Accordingly, low expression of miR-34 has been found to be correlated with unfavorable prognosis (Xie et al., 2017b). Besides, downregulation of the tumor suppressor miR-200 family has been proposed to be associated with poor prognosis in BC, and the use of this family as prognostic marker has been indicated (Wiklund et al., 2011; Martínez-Fernández et al., 2015a). The miR-200 family consists of five different members, namely miR-200a, miR-200b, miR-200c, miR-429 and miR-141, and has been suggested to play an essential role in the inhibition of the EMT process by regulation of ZEB1 and ZEB2 transcription factors (Korpal et al., 2008; Park et al., 2008).

Many other tumor suppressor and oncogenic miRNAs have been extensively described or recently discovered as particular players in BC (Enokida et al., 2016; Gulìa et al., 2017). For example, low expression of miR-100, miR-101 and miR-214, as well as high expression of miR-452, miR-21, miR-222, miR-182, miR-133b, miR-155, miR-145, and miR-152 has been correlated with unfavorable prognosis(Xie et al., 2017b).

Given their deregulated expression, the miRNAs have been widely studied as therapeutic target and biomarkers in different pathologies, including several types of cancers (Romero-Cordoba et al., 2014; Shah and Calin, 2014; Chan et al., 2015). Accordingly, the study of miRNAs in liquid biopsy offers great perspective for diagnostic and prognostic purposes. These objectives will be further discussed below.

#### Other ncRNA

CircRNA molecules represent a type of ncRNA that are covalently closed in a loop at the 3′ and 5′ ends. The lack of free 3´ or 5´ ends provides increased resistance of circRNAs to exoribonuclease-dependent RNA degradation, which results in a prolonged half-life of over 48 h (Jeck and Sharpless, 2014). Even though their cellular functions are still largely unknown, various circRNAs have shown relevance in multiple cancer types (Zhang et al., 2017; Kristensen et al., 2018). Although circRNA research in BC is still scarce, several circRNAs have been shown to be highly expressed in human BC. These endogenous circRNAs competitively target specific miRNAs, thereby suppressing miRNA activity by acting as a miRNA sponge. For example, circTCF25 has been demonstrated to promote cell proliferation and metastasis by acting as a RNA sponge for miR-103a-3p and miR-107, resulting in increased CDK6 levels (Zhong et al., 2016). Besides, circRNA-MYLK and circRNA-CTDP1 competitively bind miR-29a-3p leading to enhanced expression of its target genes *DNMT3B*, *VEGFA*, *HAS3* and *ITGB1*, resulting in angiogenesis, EMT and metastasis (Huang et al., 2016; Zhong et al., 2017). Recently, additional circRNA molecules representing an oncogenic role in BC tumorigenesis and progression have been discovered, including circCEP128, circRNA-VANGL1, circPRMT5 and circRNA-cTFRC (Chen et al., 2018; Wu et al., 2018; Zeng et al., 2019; Su et al., 2019).

Contrarily to the oncogenic role of several circRNAs, some circRNAs act as tumor suppressors and have been shown to be downregulated in human BC. For example, circRNA-ITCH has been shown to suppress the aggressive biological behavior of BC through increased expression of p21 and PTEN by sponging miR-17 and miR-224, whereas circRNA-BCRC-3 has been found to act as a sponge of miR-182-5p resulting in enhanced expression of p27 (Xie et al., 2018; Yang et al., 2018a). Other circRNA molecules which have recently been discovered to mediate anti-oncogenic functions include circRNA-BCRC4, circRNA-Cdr1as and circMTO1 (Li et al., 2017; Li et al., 2018a; Liu et al., 2018b).

Their extensive abundance, stability and tissue-specific expression make circRNAs attractive molecules for clinical research (Barrett and Salzman, 2016). Further research into their regulatory mechanisms on miRNA expression will help us to improve our knowledge regarding their function in carcinogenesis and may provide insights in the use of circRNA molecules as predictive and diagnostic biomarkers as well as novel therapeutic targets (Kulcheski et al., 2016; Han et al., 2017).

Y RNA molecules are small ncRNAs (21–24 nucleotides) necessary for DNA replication through interactions with chromatin and initiation proteins. Four Y RNAs have been identified and found to be highly evolutionary conserved, namely Y1, Y3, Y4 and Y5 (Christov et al., 2006). These ncRNAs are protected from degradation by its interaction with Ro, a ribonucleoprotein particle that provides stability to these molecules, and their abundance has been found to be relatively high (about 1 × 10^5^ molecules per cell) (Christov et al., 2006; Chen et al., 2007). Even though a role for Y RNAs in BC has been indicated by various studies, contradicting observations have been published (Christov et al., 2008; Tolkach et al., 2017). Christov et al. described the significant overexpression of two Y RNAs, Y1 and Y3, whereas Tolkach et al. published the significant downregulation of all four Y RNAs in BC tissue compared to tissue of healthy controls. Accordingly, this emphasizes the need for further studies to clarify the possible role of Y RNA in BC etiology and progression.

PiRNA molecules are short single strands non-coding RNAs (26–31 nucleotides) mediating epigenetic and post-transcriptional gene silencing through interactions with PIWI proteins (Siomi et al., 2011). Their small size suggests particular resistance to degradation, which can result in the presence of relatively high levels of piRNA molecules (Palazzo and Lee, 2015; Pardini and Naccarati, 2017). Deregulated expression of some piRNAs has been found in different cancer types (Chalbatani et al., 2019). In BC, Martinez et al. described the association of high levels of piRNA FR004819 with poorer survival, whereas Taubert et al. defined a significant association between diminished *PIWIL2* expression and poor prognosis (Martinez et al., 2015; Taubert et al., 2015). Additionally, piRABC has been observed to be downregulated in BC tissue and has been identified as an important piRNA in the development and progression of this pathology. Besides, it has been proposed that piRABC may promote cell apoptosis in BC by upregulation of the TNFSF4 protein (Chu et al., 2015; Chalbatani et al., 2019).

## Epigenetic Regulation of the Bc Microenvironment

### Immune Cell Compartment

**Figure 2:**
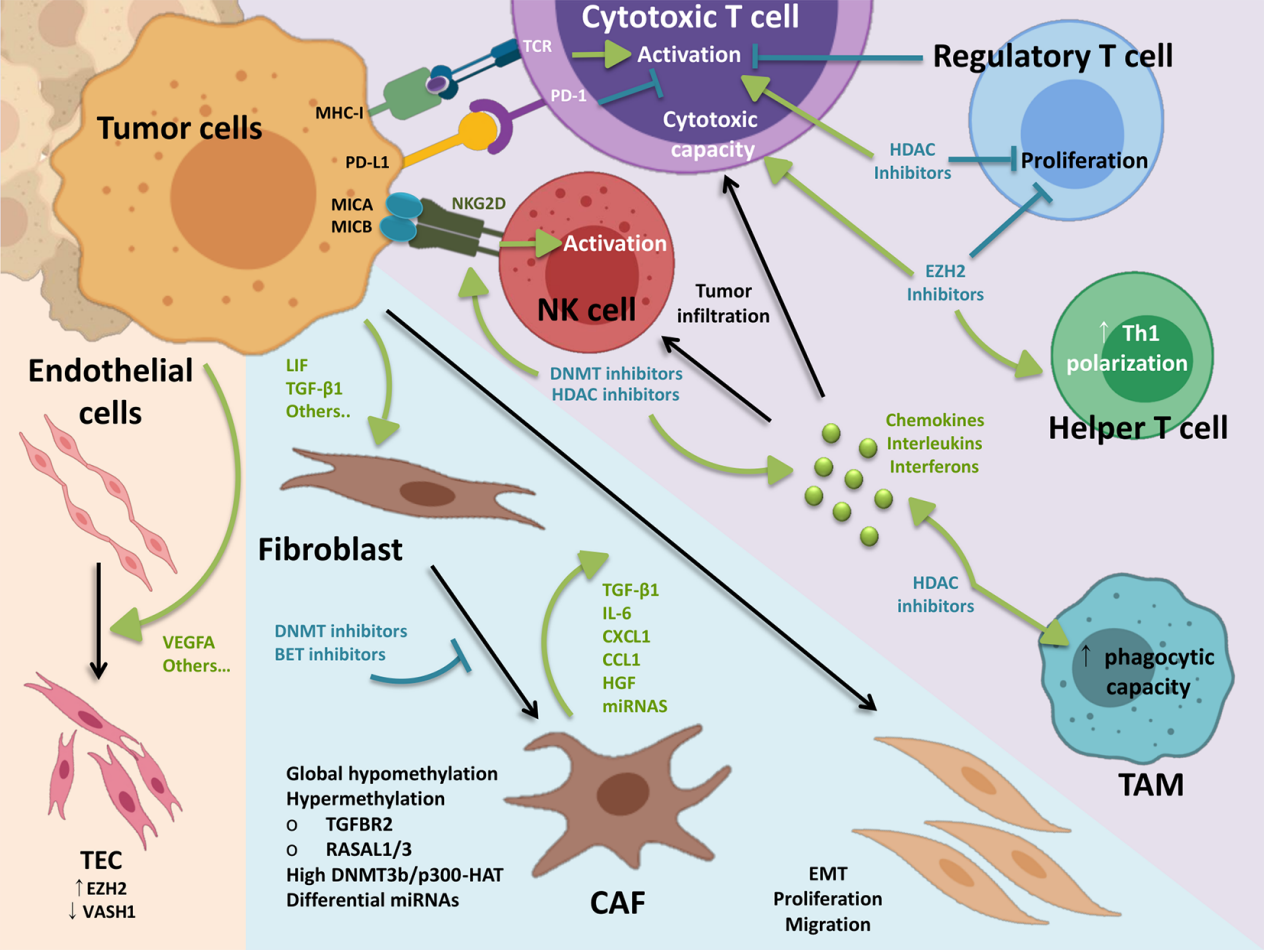
Epigenetic landscape of the tumor microenvironment. Tumor cells can influence the stroma through different factors, being soluble factors the most characterized. Tumor-derived VEGFA induces EZH2 in TEC, which drives hypermethylation of anti-angiogenic Vash1. Also induced by tumor cells, CAF differentiation is associated with several epigenetic features and can be blocked by a number of chromatin remodelers inhibitors. In turn, CAFs promote tumor growth and metastasis *via* secretion of soluble factors and matrix remodeling. On the immune side, cytotoxic T cells and natural killer cells are the main effectors of the anti-cancer immune response. Balance between activating and inhibiting signals coming from tumor targeted cells determines cytotoxic activity of these cells. Other immune cells such as regulatory T cells and macrophages are key in the anti-cancer immune response. Of note, myeloid and lymphoid lineages present inverse methylation patterns in cancer tissues, contributing to aberrant functionality. Inhibition of epigenetic writers can block regulatory T cell differentiation and function, while promoting anti-tumor activity in effector cells. Reverting tumor-driven epigenetic modifications imprinted in the TME may condition the tumor stroma for effective elimination of malignant cells in combination with existing treatments such as immunotherapy. TEC, tumor endothelial cells; CAF, cancer-associated fibroblasts; EMT, epithelial–mesechymal transition; TAM, tumor-associated macrophage.

Cancer initiation and tumor progression are often associated with the inhibition of anticancer immune response and dysregulation of inflammatory activity (Berraondo et al., 2016; Sukari et al., 2016). Different solid tumors are characterized by the presence of immune cells, such as T and B lymphocytes, natural killer (NK) cells, macrophages, and antigen-presenting cells in the tissue microenvironment (TME). These immune cells exhibit different behaviors and morphologies as a result of aberrant differentiation (Olivieri et al., 2016), sometimes driven by epigenetically regulated lineage-specific changes influencing the expression of genes crucial for the identity of immune cells and promoting cellular responses to stimuli (Herold et al., 2012; Smith and Meissner, 2013; Luperchio et al., 2014) (**Figure 2**).

Recently, some studies have shown that post-translational modification of histones may regulate the behavior of cells involved in the immune response, including tumor associated macrophages (TAMs), regulatory T cells (Tregs), dendritic cells (DCs), NK cells, myeloid-derived suppressor cells (MDSCs), effector T cells (Teffs), and others (Liu et al., 2017a). Based on whole-genome bisulfite sequencing datasets from the BLUEPRINT Epigenome Project (http://www.blueprint-epigenome.eu), Schuyler et al. identified inverse methylation patterns in the myeloid and lymphoid lineages in cancer tissues, where lymphoid-derived neoplasms lose CpG methylation patterns whereas myeloid malignancies significantly increase levels of DNA methylation (Schuyler et al., 2016). These observations have been reproduced by other authors showing that different methylation patterns contribute to the activation of myeloid and lymphoid cancer cells (Bröske et al., 2009; Bock et al., 2012).

The main component of the immune infiltrates present in solid tumors are TAMs, which have been frequently associated with worse prognosis. Compared to the binary M1/M2 classification, TAMs include multiple populations sharing features of both M1 and M2 phenotypes that in many cases do not fit the M1/M2 classification. Nonetheless, it offers a useful working frame for the study of TAMs, in which the overall consensus is that M1 macrophages are anti-tumorigenic, while M2 macrophages can promote tumor growth. M2-macrophage marker genes are epigenetically regulated by reciprocal changes in histone H3 lysine-4 (H3K4) and histone H3 lysine-27 (H3K27) methylation. After IL-4 stimulation, a decrease of H3K27 dimethylation and trimethylation (H3K27me2/3) marks occur as well as the transcriptional activation of specific M2 marker genes. Additionally to methylation, during monocyte to macrophage differentiation, there is a massive reconfiguration of lysine acetylation patterns at gene regulatory elements with a positive correlation between transcriptionally permissive H3 histone acetylation and the activity of regulatory elements (Bistoni et al., 1986).

When analyzing the activation/polarization status of tumor infiltrating lymphocytes (TILs), TAMs and DCs, several studies have shown that the methylation status of immune genes in these cells influences the tumor immune response in the TME, and correlates with the density of TILs and tumor progression. For example, in naïve CD4^+^ T cells the interferon-γ (*IFN-γ*) gene promoter and upstream enhancer is methylated. However, in Th1 lymphocytes, where the expression of *IFN-γ* is induced, the *IFN-γ* gene promoter and enhancer are demethylated, suggesting an important role in Th1/Th2 differentiation (Janson et al., 2008). The histone methyl transferase EZH2 has also been shown to play an important role in shaping the function of T cells. Wang et al. demonstrated that accumulation of H3K4me3 in the promoter of *FOXP3* results in the generation of Tregs, and pharmacological or genetic suppression of the activity of EZH2 on tumor-infiltrating Tregs (TI-Tregs) results in the acquisition of pro-inflammatory functions (Wang et al., 2018a) (**Figure 2**). In addition, suppression of EZH2 modulates the TME and enhances the infiltration of CD8^+^ and CD4^+^ effector T cells, which can favor tumor eradication (Wang et al., 2018a). Besides H3K27 methylation, G9a-dependent H3K9me2 is an important regulator of inflammatory gene expression and has also been implicated in several aspects of T cell biology. Although genome-wide studies mapping the binding of G9a (or the H3K9me2 mark) in immune cells has not been carried out, a descriptive genome-wide analysis of H3K9me2 marks in resting human lymphocytes using ChIP-on-chip methods demonstrated that this epigenetic mark is enriched on genes that are associated with several specific pathways including T cell receptor signaling, IL-4 signaling, and GATA3 transcription (Zhang et al., 2018a).

In addition to T and B lymphocytes, NK cells are effector lymphocytes of the innate immune system that have been shown to control tumor growth (Vivier et al., 2008). Although studies investigating the role of epigenetic modulation on NK cell activation and cytotoxicity are still scarce, some reports indicate that histone acetylation is involved in the regulation of NK cell activation and effector functions (Schenk et al., 2016; Raulet et al., 2017). Particularly in cancer, HDAC inhibitors have been shown to modulate the expression of NK ligands on the surface of neuroblastoma, melanoma, osteosarcoma, colon and Merkel cell (Zhu et al., 2015; Kiany et al., 2017) (**Figure 2**). Besides, Hicks et al. shows that HDAC inhibitors, in addition to significantly enhancing the expression of multiple NK ligands and death receptors resulting in enhanced NK cell-mediated lysis, also increases tumor cell PD-L1 expression both *in vitro* and in carcinoma xenografts (Hicks et al., 2018). This data offers a rationale for combining HDAC inhibitors with inhibitors of the PD-1/PD-L1 axis, including for patients who are refractory or expected not to respond to these therapies alone due to absent or low PD-L1 tumor expression.

### Cancer-Associated Fibroblasts

The tumor stroma is defined as the non-malignant cells and extracellular components that surround tumors, with a fundamental role in growth and progression. Fibroblasts in the tumor microenvironment differentiate into cancer-associated fibroblasts (CAFs), being one of the main components in the tumor stroma (**Figure 2**). CAFs play key roles in all cancerous stages, the vast majority of the studies demonstrating pro-tumoral functions that include extracellular matrix remodeling, angiogenesis, immune suppression and drug resistance (Kalluri, 2016; Tao et al., 2017; Ziani et al., 2018).

The current knowledge on CAF biology in BC is scarce and mostly coming from *in vitro* experiments. Nonetheless, it has been shown that there is a positive correlation between the presence of active CAFs and expression of EMT markers and worse prognosis in BC patients (Schulte et al., 2012; Wu et al., 2017). *In vitro*, BC cells can induce differentiation of healthy fibroblast into CAFs *via* exosomes (Ringuette Goulet et al., 2018; De Palma et al., 2019; Goulet et al., 2019) and other not fully characterized secreted factors (Wang et al., 2007; Grimm et al., 2015; Shi et al., 2015; Yeh et al., 2015). As a result, differentiated CAFs induce motility and migration in cancer cells *via* induction of EMT through secretion of a number of soluble factors which include TGF-β1 (Zhuang et al., 2015; Wu et al., 2017), IL-6 (Yeh et al., 2015; Goulet et al., 2019), and hepatocyte growth factor (HGF) (Wang et al., 2007; Grimm et al., 2015), and/or by direct chemokine attraction through CXCL1 (Shi et al., 2015) and CCL1 (Yeh et al., 2015).

Studies using global methylation analysis have shown that epigenetic modification plays a fundamental role in fibroblast activation and CAF differentiation (Hu et al., 2005; Jiang et al., 2008; Bechtel et al., 2010; Lamprecht et al., 2018). Indeed, an overall hypomethylated status was found in human CAFs (Jiang et al., 2008; Eckert et al., 2019) (**Figure 2**), as well as in functionally related fibrotic fibroblasts (Komatsu et al., 2012). Nevertheless, certain key genes appear hypermethylated in CAFs such as *Tgfbr2* (Banerjee et al., 2014), *RASAL1* and others (Bechtel et al., 2010; Zeisberg and Zeisberg, 2013; Mishra et al., 2018). Seminal work by Cedric Gaggioli’s group demonstrated that tumor-derived LIF induces activation of DNMT3b and p300-HAT in CAFs, which sustain JAK1/STAT3 signaling, necessary to maintain a pro-invasive activity (Albrengues et al., 2015). More recently, the nicotinamide N-methyltransferase has been shown as fundamental for CAF´s protumoral behavior *in vitro* and *in vivo*, directly affecting DNA and histone methylation (Eckert et al., 2019). CAF differentiation and activity *in vivo* can be blocked by treating with the DNMT inhibitor 5′-Aza-2′-deoxycytidine (Albrengues et al., 2015; Eckert et al., 2019), acting specifically in pancreatic CAFs compared to normal fibroblasts (Yu et al., 2012). Relevant results when DNMT inhibitors are considered for therapy, which will be further discussed below.

Interestingly, the RasGTP *RASAL3*, negative regulator of the Ras signaling pathway, was also found hypermethylated in prostate cancer (PCa) CAFs (Mishra et al., 2018), increasing Ras signaling in these cells, which drives support of tumor growth and neuroendocrine differentiation. Noteworthy, switch in CAFs towards a Warburg metabolism has been implicated in tumor immune evasion in PCa (Comito et al., 2019), which adds further clinical relevance of epigenetic-mediated changes in CAFs metabolism. Indeed, an *in vitro* 3D-microfluidoc system has shown that CAFs provide metabolic support to proliferation and invasion of BC cells (Shi et al., 2015). The role of epigenetic modifications in this phenomenon and its relevance *in vivo* will require further investigation.

Besides DNA methylation, other epigenetic modifications have been observed in the tumor stroma (Li et al., 2015; Du and Che, 2017; Schoepp et al., 2017; Vafaee et al., 2017; Zhao et al., 2017; Kim et al., 2018). In a proof-of-concept study, Zong et al. showed that overexpression of the non-histone chromosomal high-mobility group protein family member Hmga2 in urogenital sinus mesenchymal cells drives tumorigenesis in a model for prostatic intraepithelial neoplasia (Zong et al., 2012). In models for pancreatic cancer and *in situ* skin squamous cell carcinomas, an inhibitor of the BRD and extraterminal domain (BET) family proteins decreases tumor growth affecting specifically CAF´s secretome (Yamamoto et al., 2016; Kim et al., 2017). Since targeting histone acetylation has been proposed for combined therapy in BC (Yoon et al., 2011), it would be necessary to characterize the histone acetylation status of stromal cells in BC patients.

Many studies show that miRNAs play fundamental roles in CAF differentiation and function, a subject that has been extensively reviewed (Chou et al., 2013; Kohlhapp et al., 2015; Kuninty et al., 2016; Marks et al., 2016). MiRNAs can be expressed by CAFs or incorporated from other sources, mainly cancer cells *via* exosomes (Pang et al., 2015). The opposite is also possible, when CAFs modulate cancer cell behavior *via* transfer of miRNAs (Josson et al., 2015; Shah et al., 2015). In BC, a study compared miRNA expression between fibroblasts from healthy and tumoral human bladder, finding higher expression of miR-16 and miR-320 (Enkelmann et al., 2011). Which functions are these miRNAs regulating in CAFs and whether they can be used as surrogate markers for stroma abundance would require further investigations.

### Tumor Endothelial Cells

In solid cancers, increased *de novo* formation of blood vasculature, known as angiogenesis, is normally observed and provides adequate nourishment for the growing tumor (**Figure 2**). The link between vasculature density and worse prognosis in BC is well documented (Bochner et al., 1995). Indeed, targeting angiogenesis *via* disruption of vascular endothelial growth factor (VEGF) signaling is being considered for treating BC in combination with existing therapies (Petrylak et al., 2016; Sonpavde and Bellmunt, 2016).

Tumor endothelial cells (TECs) display a number of characteristics compared to normal endothelium (Hashizume et al., 2000; Hida et al., 2004). In BC, exacerbated proliferation and sprouting of TECs has been linked to staging and lower survival in patients (Roudnicky et al., 2013; Roudnicky et al., 2017). Invasive BC cell lines show increased adhesion to endothelial cells *via* MUC1 and CD43 binding to ICAM-1, which could be linked to metastatic potential (Laurent et al., 2014; Sundar Rajan et al., 2017). Besides, an *in vitro* study shows that TECs may promote BC cell growth through a paracrine loop involving secretion of epidermal growth factor by TECs in response to tumor-derived VEGFs (Huang et al., 2019b). Finally, TECs have been found in MIBC with aberrant expression of a non-anti-angiogenic thrombospondin-2 variant, also responsible for uncontrolled angiogenesis in these tumors (Roudnicky et al., 2018).

It is well known that epigenetic modifications play a role in endothelial cell (ECs) proliferation, differentiation and pathogenesis (Hulshoff et al., 2018; Nagai et al., 2018; Schlereth et al., 2018; Stone et al., 2018; Nicorescu et al., 2019). In fact, recent work by Wang S. and colleagues shows that response to VEGFA, a master regulator of EC biology, strongly relies on epigenetic mechanisms (Wang et al., 2019). Although less explored, several studies have addressed the role of epigenetic modifications in TECs (Marks et al., 2016). Chromatin remodeling inhibitors reduce tumor growth and angiogenesis by acting on both tumor cells (Kim et al., 2001) and ECs (Deroanne et al., 2002; Hellebrekers et al., 2006). More specifically, high expression of EZH2 in ECs is associated with high-stage and grade, and decreased overall survival in epithelial ovarian cancers (Lu et al., 2010). The authors showed that tumor-derived VEGFs induce expression of EZH2 in ECs, which in turn drives hypermethylation of the anti-angiogenic gene, *Vash1* (Lu et al., 2010) (**Figure 2**). Of note, EZH2 expression in ECs is also under control of the vascular endothelial cadherin, which appears reduced in ovarian TECs (Morini et al., 2018). As new anti-cancer therapies targeting both DNMTs and methylation readers evolve, it is necessary to evaluate their effect in TECs.

Importantly, CAF and TEC biology has a meeting point in what is known as endothelial-to-mesenchymal transition (EndMT) in cancer (Zeisberg et al., 2007). By ChIP-seq, Nagai N. and collaborators found that the transcription factor ERG/FLI1 associates with H3K27ac marks at enhancer/promoter regions of various EC-specific genes, inducing expression of miR-126, which represses EndMT genes. Using available data, the authors also found that lower expression of ERG was significantly related to poor prognosis (Nagai et al., 2018).

## New Therapies in Epigenetics

Epigenetic changes have been suggested as essential for tumor development (Biswas and Rao, 2017). As discussed before, aberrant DNA methylation, histone modifications and chromatin states, as well as aberrant expression of ncRNAs can be used as potential targets by specific drugs and combined with existing therapies. Several molecules targeting epigenetic alterations have been developed and used in different cancers. In the following section, we describe the most recent cancer drugs targeting some epigenetic enzymes. Although in most cases their applications in BC are still in its very early days, we will focus on how they are currently studied in this context.

### Drugs Targeting Writers

#### DNA Methyltransferase Inhibitors

DNMTs inhibitors (DNTMi) are classified in two major subtypes: nucleoside and non-nucleoside inhibitors. Decitabine (5-aza-2’-deoxycytidine) and Azacytidine (5-azacytidine) are cytosine analogues and the best known nucleoside DNMTi. Decitabine and Azacytidine are currently approved by FDA for the treatment of specific forms of myelodysplastic syndromes, chronic myelomonocytic leukemia and acute myeloid leukemia (Lu et al., 2011; Giagounidis et al., 2014). Regarding BC, *in vitro* experiments have demonstrated that Decitabine enhances cisplatin susceptibility, suggesting that combination of both drugs could improve clinical responses (Shang et al., 2008; Wu et al., 2019b). Moreover, Decitabine has completed phase II trials for treatment of BC and phase I trials in combination with tetrahydrouridine (Shang et al., 2008; Bertino and Otterson, 2011).

**Figure 3:**
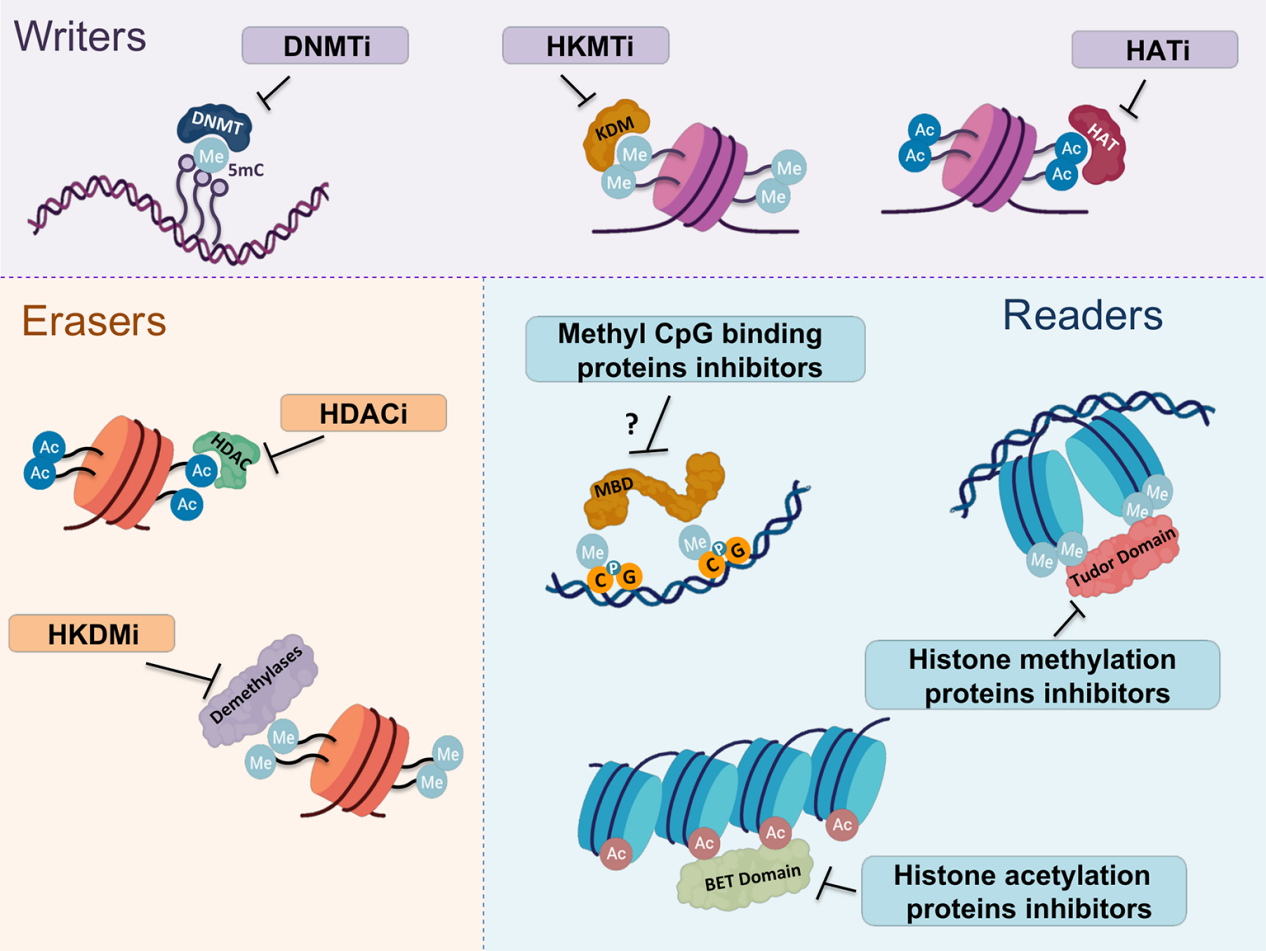
Most representative epigenetic inhibitors targeting writers, readers and erasers. Epigenetic alterations are considered to be reversible and, therefore, all these molecules are subject of study as promising therapeutic targets for cancer treatment. Three main groups of epigenetic drugs can be distinguished according to their targets. The group of compounds targeting epigenetic writers consists mainly of DNMT, HKMT and HAT inhibitors. The second group is directed against epigenetic erasers, which includes HDAC and HKDM inhibitors. Finally, inhibitors of methyl CpG binding proteins, histone methylation and acetylation proteins form the third group targeting epigenetic readers. DNMTi, methyltranferases of DNA inhibitor; HKMTi, histone lysine methyltransferase inhibitor; HATi, histone acetyltransferase inhibitor; HDACi, histone deacetylase inhibitor; HKDMi, histone lysine demethylase inhibitor.

Second generation nucleoside DNMTi, such as Guadecitabine (SGI-110) or 4’-thio-2’- deoxycytidine, have been developed in order to reduce high toxicity without reducing the therapeutic dose needed. Clinical trials for 4’-thio-2’- deoxycytidine are currently recruiting patients for the treatment of advanced solid tumors (NCT03366116). On the other hand, SGI-110 is in clinical stage for various cancers such as acute myeloid leukemia and myeloidDdysplastic syndrome (NCT03603964), and for different solid tumors like advanced hepatocellular carcinomas (NCT01752933). Also, it has been tested in combination with other therapies such as Ipilimumab in metastatic melanoma (NCT02608437) or with carboplatin in ovarian cancer (NCT01696032), among others. As immune checkpoint inhibitors are currently used in BC, their combination with these second generation DNMTi could represent an attractive scenario to improve the therapeutic response or to expand the number of patients that benfit from immunotherapy. Some others like SGI-1027, a quinoline derivative, and Nanaomycin A, a quinone antibiotic, which are reported to inhibit all three DNMTs or only DNMT3a, respectively, are in preclinical stages for colorectal cancer (Datta et al., 2009; Kuck et al., 2010) (**Figure 3** and **Table 1**).

**Table 1:** A representation of experimental epigenetic drugs targeting writers, readers and erasers.

Drugs Targeting Epigenetic Writers				
Category	Compound Name	Development Stage	Cancer Type	References
**DNA Methyltransferase inhibitors (DNMTi)**				
Nucleoside analogue	Decitabine	Approved	MDS	Giagounidis et al., 2014; Lu et al., 2011
	Azacytidine	Approved	MDS	Giagounidis et al., 2014; Lu et al., 2011
	Guadecitabine	Clinical	MDS, AML	NCT03603964
Non-nucleoside analogue	MG98	Clinical	MRCC	Winquist et al., 2006
	SGI-1027	Preclinical	Colorectal	Datta et al., 2009
	Nanaomycin A	Preclinical	Colorectal	Kuck et al., 2010
**Histone Lysine Methyltransferase inhibitors (HKMTi)**				
G9a	A-366	Preclinical	Neuroblastoma	Pappano et al., 2015
	BRD4770	Preclinical	Breast	Vedadi et al., 2012
	UNC0638	Preclinical	Leukemia	Yuan et al., 2012
EZH2	UNC1999	Preclinical	Large B-cell lymphoma	Konze et al., 2014
	GSK343	Preclinical	Glioblastoma	Yu et al., 2017
	GSK126	Clinical	Large B-cell lymphomas	NCT02082977
	EPZ6438	Clinical	B-cell Lymphomas	NCT03010982
**Histone Acetyltransferase inhibitors (HATi)**				
p300	C646	Preclinical	Gastric	Gajer et al., 2015
	PU141	Preclinical	Neuroblastoma	Wang et al., 2017
**Drugs Targeting Epigenetic Readers**				
**Histone Methylation Proteins**				
PHD Finger Domain (JARID1A)	Amiodarone	Preclinical	AML	Wagner et al., 2012
MBT Domain	UNC926	Preclinical	Target domain inhibition	Herold et al., 2012
Chromodomain (CBX7)	MS37452	Preclinical	Target domain inhibition	Ren et al., 2015
**Histone Acetylation Proteins**				
Bet Bromodomain	(+) - JQ1	Preclinical	Colorectal	Zhang et al., 2018b
	OTX015	Clinical	Advanced Solid tumors	NCT02698176
**Drugs Targeting Epigenetic Erasers**				
**Histone Lysine demethylase inhibitors (HKDMi)**				
LSD1 Inhibitors	Pargyline	Preclinical	Target domain inhibition	Yang et al., 2018b
	HCI-2509	Preclinical	Neuroblastoma	Gupta et al., 2018
JmjC Domain inhibitors	IOX1	Preclinical	Target domain inhibition	Hopkinson et al., 2013
**Histone Deacetylase inhibitors (HDACi)**				
Hydroxamic Acid Derivates	Vorinostat	Approved	CTCL	Mann et al., 2007
	Panobinostat	Approved	Blood neoplasias	Eckschlager et al., 2017
	Reminostat	Clinical	Hodgkin’s lymphoma	NCT01037478
	Quisinostat	Clinical	Ovarian cancer	NCT02948075

MDS, Myelodysplastic syndromes; MRCC, Metastasic renal cell carcinoma; AML, Acute Myeloid Leukaemia; CTCL, Cutaneous T cell-lymphoma.

Apart from these inhibitors, various non-nucleoside DNMTi have been developed and suggested to minimize the direct effect on DNA (Villar-garea et al., 2003). Non-nucleoside analogues, such as Procainamide and MG98, inhibit methylation by binding to the CpG regions of DNA and blocking the activity of DNMTs. MG98, for example, was tested against metastatic renal cell carcinoma but the clinical trial was stopped due to its toxicity (Winquist et al., 2006). However, it has also been evaluated in combination with interferon and results are promising at a specific dose (Amato et al., 2012). Moreover, MG98 was tested in BC patients but the researchers did not find response to the treatment (Plummer et al., 2009).

#### Histone Lysine Methyltransferase Inhibitors

As it was previously described in this review, HMTs such as G9a and EZH2 are considered oncogenic epigenetic factors in BC (Cho et al., 2015). One of the first histone lysine methyltransferase inhibitors (HKMTi), specific against G9a (EHMT2), was BIX-01294 (Kubicek et al., 2007), which has been shown to inhibit cell proliferation in BC cell lines and induce apoptosis in neuroblastoma cells (Cui et al., 2015). Since then, numerous and improved inhibitors related to G9a blocking have been developed. Various studies have been carried out in molecules like A-366, BRD4770 or UNC0638, in different types of cancer such as neuroblastoma, breast or leukemias (Vedadi et al., 2012; Yuan et al., 2012; Pappano et al., 2015). Recently, CM272 was described as a novel G9a/DNMT1 dual inhibitor with remarkable antitumor effect in BC *in vitro* and *in vivo* (José-Enériz et al., 2017; Segovia et al., 2019). On the same line, the catalytic subunits of PRC2, EZH1 and EZH2, which catalyze the methylation of H3K27, have been well described in cancer. Some inhibitors of this complex have been studied and they are classified into three groups: (i) pyridone-indazole scaffold like UNC1999 or GSK343 (Konze et al., 2014; Yu et al., 2017) which has been demonstrated to inhibit BC cell lines growth and metastasis (Chen et al., 2019), (ii) pyridone-indole scaffold such as GSK126 (NCT02082977) and (iii) pyridone-phenyl scaffold including EPZ6438 (Brach et al., 2017), known also as Tazemetostat, which has achieved phase I/II trial (NCT03854474) for the treatment of patients with locally advanced or metastatic urothelial carcinoma in combination with pembrolizumab. The potential use of EZH2 in the BC context has been recently reviewed and discussed (Martínez-Fernández et al., 2015c; Segovia and Paramio, 2017).

#### Histone Acetyltransferase Inhibitors

HATs are typically grouped into three broad families, namely the p300/CBP, the Gcn5 related N-acetyl-transferase and the MYST family. Among them, p300/CBP seems to be frequently mutated in BC (Duex et al., 2018a) and was reported to be associated with doxorubicin resistance (Takeuchi et al., 2012), so it could be a promising molecular therapeutic target for this disease. Accordingly, C646 and PU141 have been demonstrated to be promising in gastric cancer and neuroblastoma, respectively (Gajer et al., 2015; Wang et al., 2017). However, there is very little evidence for useful histone acetyltransferase inhibitors (HATi) being developed and tested (Baell and Miao, 2016), even though the search for new small-molecule HATi has been intense in the last decades (**Figure 3**). Although, to our knowledge no HATi are being tested in BC, it is important to consider that HAT gene deficiencies may confer susceptibilities to other inhibitors, opening new possible therapeutic approaches for various tumors, including BC (Ogiwara et al., 2016).

### Drugs Targeting Readers

#### Methyl CpG Binding Proteins

Sites of DNA methylation recruit two important protein families: MBD and ZnF proteins. The MBD protein family uses its DNA binding domains and other protein-protein domains to alter the transcriptional state of the DNA (Ginder and Williams, 2018). However, the MBD family is not the only protein family that allows the recognition of methylated DNA; for example, the Kaiso protein family (Kaiso/ZBTB33, ZBTB4 and ZBTB38) uses a three-finger zinc motif to bind methylated CGCG (Hendrich and Bird, 2015). Additionally, it has been demonstrated that ZBTB38 promotes cell migration, invasive growth and EMT in BC cell lines (Jing et al., 2018), whereas high MBD2 expression was significantly associated with reduced bladder carcinoma risk (Zhu et al., 2004). Even though different experimental approaches have identified these proteins as good therapeutic targets, inhibitors have not yet been developed to slow down their action (**Figure 3**).

#### Histone Methylation Proteins

The histone methyl protein family is a large family of proteins that binds differently to methylated lysine and arginine residues and can be divided into several subfamilies: Tudor domain, PHD finger, MBT, chromodomain and BRD. The most studied family among them is the PHD family, which comprises a group of versatile readers of the epigenome that can recognize both methylation and acetylation marks and has been involved in cancer progression (Hayami et al., 2010). Recently, Wagner et al. discovered various compounds that inhibit the PHD of this protein (Wagner et al., 2012). Among them, Amiodarone is able to induce apoptosis in the T24 BC cell line (Bognar et al., 2017). Upregulated UHRF1 (E3 ubiquitin-protein ligase 1), which contains PHDs, has also been shown to promote BC cell invasion *in vitro* and *in vivo* by epigenetic silencing of KiSS1 (Zhang et al., 2014).

#### Histone Acetylation Proteins

In general, histone acetylation is related to transcriptional activation. Different protein domains that bind specifically to acetylated histones have been identified so far, including the BRD, double PHD finger and Yeats domains. The BRD family identifies acetylated lysine residues, such as those on the *N*-terminal tails of histones, and has been proposed as an attractive therapeutic target due to its involvement in various cancer types. The BET family has been thoroughly investigated (Biswas and Rao, 2018). The first inhibitors of the BET family, I-BET762 (GSK525762) and (+)-JQ1, were reported in 2010 (Filippakopoulos et al., 2010). The inhibitor I-BET762 has recently been studied for dose escalation clinical studies to investigate the safety, pharmacokinetics, pharmacodynamics, and clinical activity in various tumors (NCT01587703), but BC patients were not included in this study. (+)-JQ1 interferes with BRD4 function, blocking the formation of the NUT-BRD4 oncoprotein, and various studies have shown its efficacy in hematological and solid malignancies (Abedin et al., 2016; Ocaña et al., 2017; Gao et al., 2018; Sakaguchi et al., 2018; Tan et al., 2018; Zhang et al., 2018b). Regarding BC, the (+)-JQ1 inhibitor induces autophagy through activation of the LKB1/AMPK pathway, contributing to the inhibition of proliferation of BC cell lines *in vitro* (Li et al., 2019). In combination with Mitomicyn C, (+)-JQ1 enhances cell death, which offers the possibility of a dose reduction of the chemotherapeutic agent (Simm et al., 2018). Hölscher et al. had also shown significant synergistic effects on the induction of apoptosis in urothelial cancer cells by treatment with (+)-JQ1 and Romidepsin, an HDAC inhibitor (HDACi), thus suggesting a promising new combination therapy approach for urothelial cancer (Hölscher et al., 2018).

Even though BRD3 inhibitors have not been studied as much as those of the BRD2/4, it has been observed that I-BET151, a pan-BET inhibitor that targets BRD3 (Picaud et al., 2013), halts the progression of the cell cycle and decreases cell proliferation *in vitro* and *in vivo* by targeting lncRNA *HOTAIR* in glioblastoma (Pastori et al., 2014). Remarkably, *HOTAIR* increased expression is also associated with poor clinical outcome in BC (Martínez-Fernández et al., 2015b), thereby indicating the possible relevance of studying I-BET151 inhibitor in this type of cancer.

### Drugs Targeting Erasers

Epigenetic marks can be ‘erased’, depending on the requirement of the cell, by a group of enzymes that oppose to the writers. Since they also modulate gene expression affecting tumor suppressor genes or oncogenes, they can be considered potential targets.

#### Histone Lysine Demethylase Inhibitors

Researchers have been exploring inhibitory molecules for the HKDMs KDM1 (LSD1) and KDM2-8 for years (Højfeldt et al., 2013). Early compounds were developed based on the structural characteristics of LSD1 (Yang et al., 2018b). Treatment with LSD1 inhibitor supressed BC cell proliferation and androgen-induced transcription, supporting a novel role for the androgen receptor-KDM (lysine demethylases) complex in BC initiation and progression (Kauffman et al., 2012). Even though numerous LSD1 inhibitors have been reported in the literature, they are in the initial phase of development and there are still many problems that have to be overcome before histone lysine demethylase inhibitors (HKDMi) can reach the clinic (**Figure 3**).

#### Histone Deacetylase Inhibitors

Various reports have shown that HDACs could be involved in regulating protein function and tumorigenesis. In this line, the use of HDACi has been clinically validated in cancer treatment and, so far, four drugs have been approved by the FDA: Vorinostat, Romidepsin, Panobinostat and Belinostat (**Figure 3**). Vorinostat was the first pan-HDACi approved by the FDA for the treatment of advanced primary cutaneous T-cell lymphoma (Mann et al., 2007). Next, various pharmaceutical companies developed other molecules such as Panobinostat or Belinostat (Eckschlager et al., 2017), all of them intended initially for blood neoplasias.

Moreover, HDACi are being studied for BC therapy (Kaletsch et al., 2018). Romidepsin and Vorinostat have been tested in a phase II trial as monotherapy, and Vorinostat has also completed phase I trials as a combination therapy with docetaxel, but it was surprisingly toxic and had limited efficacy (Cheung et al., 2008). Additionally, Belinostat has obtained positive responses in BC cells through decreasing cell proliferation *in vitro* and *in vivo* (Buckley et al., 2007) and is being tested in clinical trials against various solid tumors including BC (NCT00413322, NCT00413075).

Apart from the hydroxamic acid derivates, which are approved for the clinic, other molecules are in different phases of study. Some of them are Reminostat (4SC-201) evaluated for Hodgkin’s lymphoma (NCT01037478), Quisinostat (JNJ-26481585) for the treatment of ovarian cancer (NCT02948075), or Abexinostat (PCI-24781) which is being evaluated for sarcoma in combination with Doxorubicin (NCT01027910). **Table 1** summarizes HDACi approved and some experimental HDACi in different stages of clinical development.

Epigenetic drugs, as seen previously, have been approved as monotherapy for the treatment of different types of cancer. In addition, the combination of epigenetic drugs with standard chemotherapy or immunotherapy has been explored in recent years with promising results. The basis for this approach comes from results showing that epigenetic drugs reduce the apoptotic threshold, reverse drug resistance and/or induce immune response. Regarding BC, a large proportion of patients are not candidates to chemotherapy due to comorbidities. The use of epigenetic drugs could bring the possibility of a dose reduction, which makes these compounds attractive candidates for combination therapy for these BC patients (Witjes et al., 2014b; Fardi et al., 2018).

### Drugs Targeting ncRNAs

#### LncRNAs

Even though no lncRNA-based targeted BC treatment has been developed so far, modulation of lncRNA expression as a therapy seems promising and has already been described for other cancer types (Bhan et al., 2017). Methods described for the modulation of lncRNA expression include the use of antisense oligonucleotide (ASO) or lncRNA-specific siRNAs for transcript destabilization or degradation, as well as transcript alteration by modulation of lncRNA-encoded promotor activity. Additionally, functional disruption of lncRNAs through aptamers antagonizing the interaction with their binding partners, or the production of synthetic molecules interfering with the association between lncRNAs and regulatory factors, are possible mechanisms to modulate lncRNA expression (Bhan et al., 2017). Finally, these ncRNAs might be valuable in combination therapy and augmentation of therapeutic efficacy since modulation of their expression can enhance the therapeutic sensitivity of tumors (Bhan et al., 2017).

#### MiRNAs

There are many approaches that have been employed to silence miRNAs in cancer. These include anti-miRNA oligonucleotides (AMOs), miRNA-masking antisense oligonucleotides, peptide nucleic acids and miRNA sponges (Garzon et al., 2010). AMOs mechanism relies on the complementary base pairing of the oligonucleotide sequence to its target miRNA. Therefore, these molecules can repress cellular mRNAs involved in tumor progression and proliferation, and they can also act as competitive inhibitors of miRNAs and impair their interaction with other molecules (Lima et al., 2018b). Joana Filipa and colleagues showed that, using AMOs, they were able to silence the expression of upregulated miR-9 in a cancer cell model of gastric cancer (Lima et al., 2018a).

For BC treatment, there are some indirect therapeutic approaches that affect miRNA expression. For instance, some EZH2 inhibitors act in BC cells modulating the expression of miR-101 (Wang et al., 2014) or miR-143 (Zhang et al., 2015). However, some of these miRNAs are also induced by specific oncogenic insults in BC, indicating the potential problems of considering them as possible targets for treatment (Segovia et al., 2017).

Remarkably, a miRNA-based drug mimicking miR-34a has reached a phase I clinical trial (NCT01829971). MiRNA-34a significance in various human cancers, including BC, is increasingly recognized nowadays (Bader, 2012; Misso et al., 2014), hence the expectation in this new approach.

#### Other nCRNAs

CircRNAs and piRNAs have been described as a promising therapeutic target in multiple cancer types, including BC (see corresponding section). Potential strategies for the modulation of circRNA expression include the use of ASOs or siRNAs in order to antagonize these ncRNAs, as well as the application of the CRISPR/Cas system to partially or completely remove oncogenic circRNAs (Zhang and Xin, 2018). Regarding the modulation of piRNA expression, possible strategies include the use of synthetic piRNAs at the transcriptional and posttranscriptional level, while antibodies against PIWI proteins might be effective as a posttranscriptional approach (Assumpção et al., 2015). Nonetheless, none of these approaches are being tested in BC therapy so far.

## Epigenetic Alterations as Biomarkers in Bc: the Potential Use of Liquid Biopsy

Regarding diagnosis and surveillance of BC, a combination of cystoscopy and urine cytology is the most widely used methodology nowadays. Currently, cystoscopy is the gold standard method in clinical practice for detection and follow-up of this disease, with a sensitivity of 85–90% to detect exophytic tumors. However, this technique is highly invasive, showing a big inter-observer and intra-observer variation. On the other hand, BC urinary cytology shows a specificity of approximately 98% but a low sensitivity of 38%. The high rates of recurrence and progression of BC require continuous follow-up of patients by cystoscopy (every 3–6 months during the next 5 years) and urine cytology, making BC one of the most costly malignancies for the National Health systems of developed countries (Lodewijk et al., 2018).

For these reasons, there is a clear need to improve the current systems of diagnosis, prognosis and surveillance of BC patients. Based on the important role of epigenetic modifications in this disease, status evaluation of the involved molecules could contribute to improve these available systems. In this context, liquid biopsy has emerged as a non-invasive way to determine the genomic landscape of cancer patients, as well as to monitor treatment response, quantify minimal residual disease, and assess therapy resistance (Bardelli and Pantel, 2017; Di Meo et al., 2017; Heitzer et al., 2017; Khetrapal et al., 2018). Liquid biopsy makes reference to the sampling and assessment of biological fluids. In genitourinary cancer, due to the proximity of tumors, urine has been considered a bona fide liquid biopsy sample, being one of the most interesting samples for its easy access and collection. However, in MIBC patients after cystectomy, serum and plasma could be the most appropriate liquid biopsy samples given its invasive and metastatic character (Lodewijk et al., 2018). Currently, there are several systems to detect and follow-up BC using liquid biopsy biomarkers (including sediment cells in urine samples, CTCs in blood samples as well as RNAs and proteins in both cases), which present sensitivity and specificity values within a range of 38–98% and 65–98%, respectively (Lodewijk et al., 2018). The determination of epigenetic alterations in liquid biopsy samples, such as variation in expression levels of ncRNAs or changes in DNA methylation profiles, could improve the predictive values of the current systems of BC diagnosis, prognosis and monitoring. Next, some of the most relevant studies of epigenetic biomarkers in urine and serum/plasma samples are discussed.

### Non-Coding RNAs as Epigenetic Biomarkers in Liquid Biopsy of BC Patients

Among the different ncRNAs previously described, miRNAs have been the most widely studied molecules in liquid biopsies so far. MiRNA molecules have several characteristics which make them potential candidates as good biomarkers in liquid biopsy samples: i) they show very homogeneous expression levels among individuals and specific expression profiles in different types of tissue (Liang et al., 2007); ii) they are included in a protein complex and, usually, in exosomes, which confers them high stability, preserving their integrity and preventing their degradation (Weber et al., 2010; Ge et al., 2014; Martínez-Fernández et al., 2016); iii) there are several systems designed to determinate ncRNA expression using RT-qPCR, which allow evaluating a large number of miRNAs from very small amounts of total RNA and at a low cost.

Given the potential of miRNAs, many studies have evaluated their predictive properties, individually or in combination, in the urine of BC patients. In this context, high expression levels of miR-146a-5p and miR-106b have been related with invasion and high grade and stage BC (Zhou et al., 2014; Sasaki et al., 2016). NMIBC patients present high levels of miR-214 in urine samples and, curiously, expression of this miRNA was inversely correlated with risk of recurrence of BC patients (Kim et al., 2013). Besides, some miRNAs such as miR-92a-3p and miR-140-5p have been associated with progression after recurrence (Ingelmo-Torres et al., 2017). Yun and collaborators have demonstrated that urine miR-145 expression levels decrease in BC patients with respect to healthy controls, both in non-invasive and invasive tumors (77.8% and 84.1% sensitivity, respectively, and 61.1% specificity in both cases). They observed an association between downregulation of miR-200a and high risk of recurrence in patients with invasive tumors (Yun et al., 2012). Besides, miR-155 has proved to be a good biomarker in urine samples, distinguishing non-invasive tumors, inflammation and healthy controls with a sensitivity of 80.2% and a specificity of 84.6% (Zhang et al., 2016).

As previously mentioned, detection of miRNA deregulation in serum or plasma may have special relevance in invasive and metastatic tumors. Yang and colleagues observed miR-210 increased expression levels in serum samples of BC patients, being associated with tumor stage, grade, and useful to predict tumor progression (AUC = 0.898) (Yang et al., 2015). Moreover, some studies using plasma have found a positive correlation between upregulation of miR-19a and miR-200b with tumor grade and stage respectively, whereas miR-92 and miR-33 presented inverse association with tumor stage (Adam et al., 2013; Feng et al., 2014).

In recent years, several panels of miRNAs (encompassing profiles from 6 to 25 miRNAs) have been developed in both urine and serum for BC diagnosis, prognosis and monitoring of recurrence. In this context, we have recently gathered some of the main miRNA profiles in BC liquid biopsies which can be consulted in Table 2 at Lodewijk et al. (2018).

Although variation in lncRNA expression levels has not been studied as widely as miRNAs in liquid biopsy samples, altered levels of expression of these molecules have been found in urine and blood samples of BC patients. Increased expression levels of *UCA1* in urine samples has been associated with the presence of high-grade NMIBC, and an integrative meta-analysis including more than 500 BC patients and healthy donors determined that its upregulation may predict BC (81% sensitivity and 86% specificity, AUC = 0.88) (Wang et al., 2006; Cui et al., 2017). In addition, other lncRNAs such as *HOTAIR*, *MALAT1*, *HOX-AS-2*, *OTX2-AS1*, *HYMAI*, *LINC00477* and *LOC100506688*, have shown upregulation in urine exosomes of MIBC patients (Berrondo et al., 2016). In addition, *H19* gene expression is significantly higher in BC patients, and its presence has been detected in the urine of 90.5% of patients *versus* 25.9% of healthy controls (AUC = 0.933) (Gielchinsky et al., 2017).

Additionally, other ncRNAs such as piRNAs and circRNA have been evaluated in biofluids. Both molecules have shown a particular resistance to degradation by exoribonuclease, making them ideal candidates for biomarker development (Pardini and Naccarati, 2017; Vo et al., 2019). Several studies have reported that piRNAs are widely detected in liquid biopsy samples, being especially abundant in urine samples and, therefore, good candidates as new biomarkers for BC. Although no deregulated piRNAs have been found in urine or blood of BC patients so far, expression level alterations of some of these molecules have been shown in liquid biopsy samples of other tumor types (Freedman et al., 2016; Iliev et al., 2016; Yuan et al., 2016; Pardini and Naccarati, 2017). Regarding circRNA, Vo and collaborators have recently developed MiOncoCirc, a technology based on exome capture RNA-seq, which stands as the first cancer-focused circRNA resource to facilitate the study of circRNAs as new biological markers of cancer (Vo et al., 2019). They were able to identify candidate circRNAs which could serve as biomarkers for prostate cancer, detecting circRNAs in urine (Vo et al., 2019). This technology could open new possibilities to find new biomarkers with predictive values in liquid biopsy samples of BC patients. Nevertheless, even though circRNAs show great potential as valuable biomarker in urine, these RNAs seem to be highly susceptible to circulating RNA endonucleases showing a half-life of only 15 seconds in human serum, which limits their use as a biomarker in this biological fluid (Jeck and Sharpless, 2014).

### DNA Methylation Profiles as Epigenetic Biomarkers in Liquid Biopsy of BC Patients.

As mentioned above, changes in methylation are chemically stable and have been broadly reported in BC. Therefore, they are an interesting source of candidate biomarkers to be detected in biofluids including both blood and urine. Currently, there are multiple methods for detecting changes in methylation comprising global genome methylation and specific genes of interest assays. The majority of methods to evaluate specific genes are based on bisulfite conversion followed by PCR and sequencing, pyrosequencing or methylation-specific PCR, among others, which generally show a high sensitivity and specificity and low assay-to-assay variability (Kurdyukov and Bullock, 2016). Already in 2002, Valenzuela and collaborators found that methylation in *p16(lNK4a)* promoter in serum could be useful as diagnostic biomarker with 22% of sensitivity, 95% of specificity and a positive predictive value of 0.98 (Valenzuela et al., 2002). Also in serum, both the methylation in promoters of protocadherin 17 (*PCDH17*) and protocadherin-10 (*PCDH10*) showed an association with BC poor prognosis (Lin et al., 2012; Luo et al., 2014). A slight association between hypermethylation in *p16(lNK4a)* and *DAPK* promoter regions and NMIBC has been also described (Jabłonowski et al., 2011). Finally, the presence of hypermethylated DNA in *APC*, *GSTP1* or *TIG1* in the serum of BC patients was associated with a worse outcome showing 80% sensitivity and 93% specificity for BC detection (Ellinger et al., 2015).

Important for BC, alterations in DNA methylation can be also assessed both in circulating cell-free DNA and in cells shed into urine. In general, it seems that a prevalence of hypermethylated genes is found in urine from BC patients. For instance, the evaluation of methylation in *TWIST1* and *NID2* in urine sediment has shown 90% sensitivity and 93% specificity (Renard et al., 2010; Fantony et al., 2017; van der Heijden et al., 2018). Other studies showed promising results using methylation of *CFTR*, *SALL3* and *TWIST1* genes in urine cell pellets in combination with cytology (van der Heijden et al., 2018). Interestingly, *SOX-1*, *IRAK3*, and *Li-MET* genes methylation status has showed better recurrence predictivity than urine cytology and cystoscopy (80 vs. 35 vs. 15%) (Su et al., 2014). Also in urine sediments, methylation in *p14ARF*, *p16INK4A*, *RASSF1A*, *DAPK*, and *APC* showed a correlation with BC grade and stage (Pietrusiński et al., 2017). Guo et al. used the methylation status for *VIM*, *RASSF1A*, *GDF15*, and *TMEFF2* to identify BC with 82% sensitivity and 53% specificity (Li et al., 2018b). *RBBP8* has been identified as almost exclusively hypermethylated in BC (Mijnes et al., 2018), while Chen et al. showed *CDH13* methylation as a biomarker with prognostic value for BC screening in urine samples (Ren et al., 2016). Using quantitative methylation-specific PCR, a novel two-gene panel with high accuracy in an urine-based test has just been described (Bosschieter et al., 2019). When stratifying in low- or high-risk NMIBC patients, 97.6% sensitivity and 84.8% specificity were obtained using promoter hypermethylation of *HS3ST2*, *SEPTIN9* and *SLIT2* genes in combination with *FGFR3* mutation (Roperch et al., 2016). Interestingly, Patchsung et al. obtained a sensitivity and specificity of 96% for BC screening using a combination of the urinary hypomethylated *LINE-1* loci and the plasma protein carbonyl content (Patchsung et al., 2012). But methylation value has not only been studied in genes and their promoters: for example, last year Shindo et al. reported a study using the methylation of four miRNAs (miR-9-3, miR-124-2, miR-124-3, and miR-137) in voided urine samples, finding an association with recurrence and radical cystectomy (Kitajima et al., 2017).

As a consequence of these new results, there are currently several clinical trials using promising urine-based tests. Among them, Bladder EpiCheck™ (based on the use of methylation-sensitive restriction enzymes followed by RT-PCR) includes a panel of 15 DNA methylation patterns for the identification of recurrent BC from urine samples. First validation results with data from 357 patients showed 88% specificity and a negative predictive value (NPV) of 94.4% for the detection of any cancer, and a NPV of 99.3% for the detection of high-grade cancer (D’Andrea et al., 2019). Another test is AssureMDx, which uses methylation of *OTX1*, *ONECUT2* and *TWIST1* in addition to mutational load of *FGFR3*, *TERT* and *HRAS* in cell pellets from urine samples, showing a sensitivity of 93–97% and a specificity around 81.7–86% (van Kessel et al., 2017). Finally, Uromark was described 2 years ago as a targeted bisulfite next-generation sequencing assay based on 150 CpG loci to diagnose BC from urine with a sensitivity of 98%, specificity of 97% and NPV of 97% for the detection of primary BC (Feber et al., 2017). Following these results, DETECT I and DETECT II are two multi-centre prospective observational studies designed to conduct a robust validation of the UroMark assay. DETECT I will recruit patients having diagnostic investigations for haematuria, while DETECT II will recruit patients with new or recurrent BC to determine respectively the NPV and the sensitivity of UroMark.

As a conclusion, although validation studies are still ongoing, the recent and promising results prompt us to be optimistic and have confidence in a near clinical implementation of a urine methylation test for BC diagnosis and prognosis.

## Future Prospects

It is clear that epigenetics has reshaped most of our concepts of biology and, undoubtedly, molecular biology understanding of human pathologies. From the point of view of those researchers interested in BC, or even in cancer in general, it is almost impossible to predict what the future will bring us in this field, but there are two clear emerging facets at our hands. On the one hand, the use of compounds interfering with many epigenetic processes combined with other therapies currently in the clinics and, on the other hand, the use of these therapies directed not only towards the tumor cells, but also the tumor niche. Obviously, from our current knowledge of immunotherapies, there is a faint border between these two concepts.

Epigenetic drugs, as seen in the previous sections, have been approved as monotherapy for the treatment of different types of cancer. Additionally, they have been shown to synergize with other epigenetic substances or anticancer therapies. The first preclinical investigations focused on the combination of DNMTi and HDACi (Cameron et al., 1999). After a while and due to the development of new epigenetic agents directed to other targets such as HMTs, HDMs or BRDs, new synergistic combinations with DNMTi and/or HDACi are being explored. In addition, due to the importance of immunotherapy in cancer, the combination of epigenetic drugs with standard chemotherapy or immunotherapy has also increased in recent years (Dunn and Rao, 2017). This is based on the theory of using epigenetic drugs to reduce the apoptotic threshold, reverse drug resistance or induce immune responses for further treatment such as chemotherapy or immunotherapy. The concept of partnering epigenetic therapy with reshaping stromal component strategies has generated a wave of translational research that highlights the potential for this approach in many different cancer types. Epigenetic drugs such as DMNTi and HDACi can reverse immune suppression, and modulate stromal cells and extracellular matrix *via* several mechanisms such as enhancing expression of tumor-associated antigens, components of the antigen processing and presenting machinery pathways, immune checkpoint inhibitors, chemokines, and other immune-related genes, as well as changing the CAFs secretomes that will favor or impede the tumor growth. But deep studies of each component interaction are still in their early days. The discoveries in these areas have established a highly promising basis for studies using combined epigenetic and immunotherapeutic agents as anti-cancer therapies with expected long lasting antitumor responses.

Finally, new areas of research such as the use of new gene targeting strategies as therapeutic tools or the potential role of epigenetic mechanisms leading to altered glycosylation, which may clearly impact the liquid biopsy and immunotherapy fields (Dall’Olio and Trinchera, 2017), may represent new horizons in BC management and detection.

## Author Contributions

All authors contributed equally to review the current literature and write specific sections. The whole work was coordinated by JP. All the authors agreed with the final version.

## Funding

This study was funded by the following: FEDER cofounded MINECO grant SAF2015-66015-R, grant ISCIII-RETIC RD12/0036/0009, PIE 15/00076 and CB/16/00228 to JP. VM is funded by Consejería de investigación e Innovación, Comunidad de Madrid (ref 2018-T2/BMD-10342).

## Conflict of Interest

The authors declare that the research was conducted in the absence of any commercial or financial relationships that could be construed as a potential conflict of interest.
